# Identification of Podoplanin Aptamers by SELEX for Protein Detection and Inhibition of Platelet Aggregation Stimulated by C-Type Lectin-like Receptor 2

**DOI:** 10.3390/bios14100464

**Published:** 2024-09-27

**Authors:** Hui-Ju Tsai, Kai-Wen Cheng, Jou-Chen Li, Tsai-Xiang Ruan, Ting-Hsin Chang, Jin-Ru Wang, Ching-Ping Tseng

**Affiliations:** 1 Department of Medical Biotechnology and Laboratory Science, College of Medicine, Chang Gung University, Taoyuan 33302, Taiwan; 2 Department of Pharmacology, College of Medicine, Chang Gung University, Taoyuan 33302, Taiwan; 3 Graduate Institute of Biomedical Science, College of Medicine, Chang Gung University, Taoyuan 33302, Taiwan; 4 Department of Laboratory Medicine, Chang Gung Memorial Hospital, Linkou Branch, Taoyuan 33302, Taiwan

**Keywords:** aptamer, C-type lectin-like receptor 2, platelet aggregation, podoplanin, systematic evolution of ligands by exponential enrichment

## Abstract

Tumor cell-induced platelet aggregation (TCIPA) is a mechanism for the protection of tumor cells in the bloodstream and the promotion of tumor progression and metastases. The platelet C-type lectin-like receptor 2 (CLEC-2) can bind podoplanin (PDPN) on a cancer cell surface to facilitate TCIPA. Selective blockage of PDPN-mediated platelet–tumor cell interaction is a plausible strategy for inhibiting metastases. In this study, we aimed to screen for aptamers, which are the single-stranded DNA oligonucleotides that form a specific three-dimensional structure, bind to specific molecular targets with high affinity and specificity, bind to PDPN, and interfere with PDPN/CLEC-2 interactions. The systematic evolution of ligands by exponential enrichment (SELEX) was employed to enrich aptamers that recognize PDPN. The initial characterization of ssDNA pools enriched by SELEX revealed a PDPN aptamer designated as A1 displaying parallel-type G-quadruplexes and long stem-and-loop structures and binding PDPN with a material with a dissociation constant (K_d_) of 1.3 ± 1.2 nM. The A1 aptamer recognized both the native and denatured form of PDPN. Notably, the A1 aptamer was able to quantitatively detect PDPN proteins in Western blot analysis. The A1 aptamer could interfere with the interaction between PDPN and CLEC-2 and inhibit PDPN-induced platelet aggregation in a concentration-dependent manner. These findings indicated that the A1 aptamer is a candidate for the development of biosensors in detecting the levels of PDPN expression. The action by A1 aptamer could result in the prevention of tumor cell metastases, and if so, could become an effective pharmacological agent in treating cancer patients.

## 1. Introduction

Tumor progression (cancer cachexia and distant metastasis) and cancer-associated thrombosis are the main causes of death in cancer patients. Most cancer cells disseminated into the bloodstream are rapidly eliminated by high-shear blood flow and the host immune system. Only a few cancer cells (<0.1%) detached from the primary tumor site survive in the blood circulatory system and cause metastasis [1]. Tumor cell-induced platelet aggregation (TCIPA) refers to the process by which cancer cells trigger the aggregation of platelets. This aggregation is facilitated by the ability of cancer cells to generate or express specific molecules or surface receptors that interact with platelets. TCIPA plays a critical role in enhancing the survival of tumor cells within the bloodstream, which is important for the distant metastasis of cancer [1,2]. Various agents, such as aspirin, apyrase, and Abciximab, have been developed for blocking cancer metastases by inhibiting tumor cell–platelet interactions [3,4,5,6]. However, agents that target platelet hemostatic proteins are often associated with an increased risk of bleeding [7,8,9,10]. It is crucial to develop anti-TCIPA agents that do not interfere with physiological hemostasis for their utility as an anti-cancer therapeutic regimen. 

Podoplanin (PDPN) is a membranous sialoglycoprotein and is among the most frequently upregulated genes in squamous cell carcinoma, central nervous system tumors, and germinal neoplasia [11,12]. PDPN binds to the C-type lectin-like receptor 2 (CLEC-2) of platelets [13,14], subsequently triggering the tyrosine phosphorylation of Src family kinases, Syk, and phospholipase C gamma 2 (PLCγ2) and inducing platelet aggregation [15,16,17,18,19]. PDPN has been shown to enhance the formation of metastatic foci, tumor progression, and cancer-associated thrombosis in animal studies without direct effects on tumor growth [13]. The potency of cancer metastasis and the embolization of cancer cells in the microvasculature of a lung are highly related to PDPN-induced platelet aggregation [13,20,21]. Based on the findings that CLEC-2-deficient platelets elicit normal responses to platelet agonists, such as protease-activated receptor 4 peptide, ADP, collagen, and the thromboxane A_2_ agonist U46619 [22], the specific inhibition of CLEC-2 signaling is postulated to have no effects on physiological hemostasis. Hence, the PDPN/CLEC-2 axis is a potential therapeutic target for anti-cancer metastasis and anti-cancer-associated thrombosis.

Various approaches for the blockage of the PDPN/CLEC-2 signaling axis and PDPN-induced platelet–tumor cell interactions have been developed. Anti-PDPN monoclonal antibodies have been used to attenuate PDPN/CLEC-2 interactions in several preclinical studies. The anti-PDPN antibodies NZ-1 and NZ-8 induce antibody- and complement-dependent cellular cytotoxicity, resulting in the removal of cancer cells and the suppression of TCIPA, tumor growth, and tumor metastases [20]. Cancer growth and metastases are prevented when mice are treated with the chimeric anti-PDPN antibodies ChMS-1 and hP2-0 [21]. The production of these antibodies is usually costly. Interference of PDPN/CLEC-2 interactions by the small molecule inhibitors 2CP and cobalt–hematoporphyrin suppresses platelet activation and cancer-associated thrombosis [22,23,24]. The application of PDPN inhibitors to experimental mice in a mouse model of occlusion-induced stroke attenuates pathological changes after cerebral ischemia-reperfusion [25]. The major drawbacks of these inhibitors are their poor solubility in aqueous solution, their low affinity, the lack of oral availability, and the toxicity of both compounds [26]. The interactions of PDPN, CLEC-2, and TCIPA are blocked by the polysaccharide-containing fraction from Artemisia argyi [27]. However, the effective ingredient has not yet been defined. There is an unmet need to identify new regimens that can be used for measuring PDPN protein expression and for interfering with both PDPN/CLEC-2 interactions and PDPN-induced platelet aggregation.

Aptamers, also termed “chemical antibodies”, provide an option for interfering with PDPN/CLEC-2 interactions. Aptamers are either single-stranded DNA or RNA oligonucleotides that form a specific and stable three-dimensional structure and bind to specific molecular targets with high affinity and specificity [28]. The “Systematic Evolution of Ligands by Exponential Enrichment (SELEX)” method is a powerful technique used to select aptamers from libraries containing random oligonucleotides of up to 10^16^ unique sequences. Through the processes of binding, separation, amplification, and enrichment, the SELEX method allows researchers to identify aptamers that specifically bind to a target molecule, such as a protein, small molecule, or even whole cells [29]. Aptamers, with high affinity and specificity to targets, have promising applications in therapy and diagnostics [30]. For example, the Thrombin Binding Aptamer (TBA) is one of the most investigated therapeutic aptamers that binds tightly to thrombin and has considerable anticoagulant activity [31]. An FDA-approved anti-VEGF aptamer, Pegaptanib, is effective for the treatment of age-related macular degeneration [32]. Aptamers against cellular signaling molecules associated with cancer or neurodegenerative diseases, as well as cancer cell-derived small extracellular vesicles, have been demonstrated as potential diagnostic and therapeutic agents [33,34,35]. Compared to antibodies, aptamers are minimally immunogenic, can be produced in a cell-free chemical synthesis system, and are less expensive.

This study aims to identify PDPN aptamers through the SELEX screening of a library of random oligonucleotides. A novel PDPN aptamer A1 is identified and defined to elicit inhibitory activity on PDPN/CLEC-2 interactions and PDPN-induced platelet aggregation. The significance of these findings is discussed. 

## 2. Materials and Methods

### 2.1. Materials

All oligonucleotides were purchased from Integrated DNA Technologies (Coralville, IA, USA). The PDPN and CLEC-2 recombinant proteins were purchased from Sino Biolgocial (Houston, TX, USA). The rat anti-human PDPN antibody (NZ-1) was purchased from AngioBio (San Diego, CA, USA). The goat anti-CLEC-2 antibody (AF1718) and mouse anti-human CLEC-2 antibody (MAB1718) were purchased from R&D Systems (Minneapolis, MN, USA). The anti-histidine antibody was purchased from Bio-Rad (Hercules, CA, USA). Horseradish peroxidase (HRP)-conjugated streptavidin was purchased from GE Healthcare (Piscataway, NJ, USA). Streptavidin sepharose beads and all chemical compounds were purchased from Sigma-Aldrich (Saint Louis, MO, USA). The ssDNA/RNA clean and concentrator was purchased from Zymo Research (Irvine, CA, USA). The NEBNext Ultra II DNA Library Prep Kit (NEB #E7645S) was purchased from New England Biolabs (Ipswich, MA, USA). 

### 2.2. Systematic Evolution of Ligands by Exponential Enrichment (SELEX) 

SELEX was performed to screen for PDPN aptamers as previously described with some modifications [36]. Briefly, recombinant PDPN was added to each well of an 8-well strip and incubated overnight at 4 °C, followed by blocking with 1% bovine serum albumin (BSA). The wells were washed three times with 1× phosphate-buffered saline (PBS) supplemented with 0.05% Tween-20 and three times with binding buffer A (1× PBS containing 5 mM MgCl_2_, 2 mM CaCl_2_, 1 mg/mL BSA, and 0.1 mg/mL salmon sperm DNA). The ssDNA library (5′-ATCCAGAGTGACGCAGCA-N40-TGGACACGGTGGCTTAGT-3′) was heated at 95 °C for 5 min and immediately placed on ice. The cooled ssDNA library was added to each well and incubated at room temperature for 1 h. The supernatant (unbound ssDNA) was first removed and then washed and eluted with water at 95 °C for 5 min. The eluted ssDNA was amplified by PCR with forward primer (5′-ATCCAGAGTGACGCAGCA-3′) and biotin-conjugated reverse primer (5′-biotin-ACTAAGCCACCGTGTCCA-3′). The streptavidin sepharose beads were added to the column, followed by washing with 1× PBS prior to the addition of the PCR products. The sense template of the amplified ssDNA was eluted from the column using 200 mM NaOH followed by purification using ssDNA/RNA clean and concentrator. SELEX was performed for nine consecutive rounds. 

### 2.3. Next-Generation Sequencing (NGS) and Data Processing

The sequences of the final PCR products from SELEX were analyzed by NGS through the NGS Core Facility at Chang Gung University. Library preparation was performed using the NEBNext Ultra II DNA Library Prep Kit following the manufacturer’s instructions. Briefly, a total of 100 ng of PCR product was end-repaired and A-tailed. The prepared DNA fragments were ligated at their 5′ ends with a sequencing-specific adapter. After library amplification and size selection, the DNA library templates were analyzed with the Agilent Bioanalyzer 2100 to evaluate the library quality. The qualified libraries were sequenced using NextSeq 500 (Illumina, San Diego, CA, USA) with a High Output Kit V2 (2*75 paired end) (Illumina, San Diego, CA, USA) according to the manufacturer’s protocol. 

The NGS sequencing data were analyzed according to the following procedures. Briefly, the low-quality reads with bases after Ns were removed. The sequences corresponding to the Illumina adapter and the universal primer region of the library were identified and trimmed from the reads. The remaining sequences of each read with a length of 40 nucleotides after processing were retained for further analysis. The frequency of unique reads was counted, and the most frequent 1000 unique sequences were output in an Excel file for further analysis. The top 100 unique sequences were aligned using BioEdit Version 7.0.5.3 software (Ibis Biosciences, Carlsbad, CA, USA). The sequences were aligned prior to searching for the conserved regions with the maximum averaged entropy being set to 0.3 without setting the maximum entropy per position. The putative 2-D structure of the aptamer was predicted by the mFold web server (http://www.unafold.org/mfold/applications/Structure-display-and-free-energy-determination.php) (accessed on 27 August 2024) [37] and by the NUPACK Web application (https://www.nupack.org/analysis/input) (accessed on 15 September 2022) [38,39].

### 2.4. Dot Blot Analysis

For the dot blot analysis of PDPN/CLEC-2 binding, a recombinant PDPN protein was spotted on a nitrocellulose membrane and air-dried for 15 min. After blocking using 5% dry milk, different concentrations of recombinant CLEC-2 protein were applied to the membrane and incubated for 1 h followed by several washes with 1× PBS containing 0.1% Tween-20 (PBST). The goat anti-CLEC-2 primary antibody was then applied to the membrane and incubated for 1 h. After several washes with 1× PBST, the membrane was incubated with the HRP-conjugated donkey anti-goat secondary antibody. After three washes with 1× PBST, CLEC-2 binding to the PDPN protein spotted on the membrane was determined by the enhanced chemiluminescence (ECL) method.

For the analysis of PDPN–aptamer binding, the nitrocellulose membrane spotted with recombinant PDPN protein was incubated with the indicated biotin-labeled aptamers in the binding buffer B (5 mM MgCl_2_, 0.1% BSA, and 0.01% yeast tRNA in 1× PBS). After several washes with the washing buffer (5 mM MgCl_2_ and 0.1% BSA in 1× PBS), the membrane was incubated with HRP-conjugated streptavidin. After three washes with washing buffer, aptamer binding on the PDPN protein was determined by the ECL method.

### 2.5. Bio-Layer Interferometry Assay 

Bio-layer interferometry (BLI) assay was performed using the Octet RED 96 system (ForteBio, Fremont, CA, USA) with some modifications [40]. Briefly, the ssDNA was labeled with biotin by asymmetric PCR amplification using a biotin-conjugated forward primer and reverse primer. The PCR products and the indicated concentrations of PDPN proteins were placed in the wells of a 96-well plate, respectively. Streptavidin-coated biosensor tips were coated with the biotin-conjugated aptamer by subsequently mounting and dipping the biosensor tips into the wells containing binding buffer B for 60 s, biotinylated PCR products for 300 s, and the binding buffer A again for 60 s. The biosensor tips coated with the aptamers were then dipped into the wells containing respective concentrations of PDPN proteins. The wavelength shift, which indicates the binding between aptamer and PDPN, was recorded for 600 s. This was followed by incubation in the binding buffer for an additional 600 s to acquire the dissociation signals.

### 2.6. Plasmid Construction

The plasmid of pcDNA4-hCLEC-2, which contained FLAG-tag, myc-Tag, and His-Tag at the C-terminus of the human CLEC-2 coding sequence, was generated by the insertion of a 775-bp PCR fragment containing hCLEC-2-FLAG into the pcDNA4-myc-His-A plasmid (5063 bp) digested with *Eco*R I and *Eco*R V. For the generation of pcDNA4-hCLEC2-no Tag, the plasmid of pcDNA4-hCLEC-2 was disgested with *Cla* I and *Pme* I to remove all the tag sequences at the 3′-end of hCLEC-2 cDNA sequences. Klenow enzyme was then used to fill in the sticky end, and the plasmid pcDNA4-hCLEC2-no Tag was generated after ligation by T4 DNA ligase. For the generation of pcDNA4-hCLEC2-His-Tag, the plasmid of pcDNA4-hCLEC-2 was digested with *Cla* I and *Age* I to remove the tag sequences except for the His-Tag. The pcDNA4-hCLEC2-His-Tag plasmid was then generated after filling in with Klenow enzyme and ligation by T4 DNA ligase. 

### 2.7. Transient Transfection

HEK293T cells were cultured in DMEM medium, which was supplemented with 10% fetal bovine serum (FBS) and 1% penicillin/streptomycin (10% DMEM). HEK293T cells (1 × 10^6^) were seeded in a 3.5 cm dish overnight. HEK293T cells were transfected with 6 μg of plasmid and 10 μL of Lipofectamine 2000 reagent in Opti-MEM medium. At 6 h after transfection, Opti-MEM medium was removed and replaced with 10% DMEM. After 18 h, transfected 293T cells were collected for Western blot analysis or flow cytometry analysis.

### 2.8. Western Blot Analysis

SDS-PAGE electrophoresis was performed using 12% polyacrylamide gels with a running voltage of 80 V for 3 h. For Western blot analysis, proteins were transferred onto the PVDF membrane, which was incubated with 5% skimmed milk at 4 °C to block the non-specific binding of the antibodies. The membrane was then incubated with the mouse anti-histidine antibody (1:20,000), the goat anti-CLEC-2 antibody (1:1000), or the biotin-conjugated PDPN A1 aptamer (200 nM) for 1 h, followed by three 10 min washes with 0.01% PBST. The HRP-conjugated goat anti-mouse antibody (1:20,000), HRP-conjugated rabbit anti-goat antibody (1:5000), or HRP-conjugated streptavidin (1:2000) was then applied for 1 h. After washing with 0.01% PBST three times, a mixture of luminol and peroxide was added to the membrane and incubated for 1 min to generate a luminescence signal from the target protein that was bound with the secondary antibody or the HRP-conjugated streptavidin. To visualize the signal on film, an autoradiography was performed in a darkroom.

### 2.9. Flow Cytometry Analysis

Flow cytometry analysis was conducted as previously described with some modifications [41]. Briefly, transfected HEK293T cells (5 × 10^5^/100 μL 10% DMEM medium) were incubated with 2.5 μL of the mouse anti-human CLEC-2 antibody (0.5 mg/mL) at 4 °C for 1 h and then washed twice with 1 mL of medium. After washing, transfected HEK293T cells were resuspended in 0.1 mL of medium and then stained with 1.5 μL of FITC-conjugated goat anti-mouse IgG antibody (0.5 mg/mL) at 4 °C for 1 h. After washing twice with 1 mL of medium, transfected HEK293T cells were resuspended in 0.2 mL of medium and subjected to flow cytometry using an Accuri C6 Flow Cytometer with CFlow^®^ Software version 1.0.264.21 (BD Biosciences, Franklin Lakes, NJ, USA). 

For the analysis of the characteristics of PDPN-A1 binding to the cells, 2 μL of FAM-labeled A1 aptamer (100 μM) was added into 48 μL of binding buffer B and incubated at 85 °C for 5 min. The FAM-labeled A1 aptamer was then refolded on ice for 10 min. HEK293T cells, PDPN^+^-OECM-1 cells, and CHO-K1 cells (2 × 10^5^/100 μL of binding buffer) were incubated with the indicated refolded FAM-labeled A1 aptamer at 4 °C for 30 min. After washing twice with 1 mL of medium, cells were resuspended in 0.1 mL of medium and subjected to flow cytometry using an Accuri C6 Flow Cytometer with CFlow^®^ Software version 1.0.264.21 (BD Biosciences, Franklin Lakes, NJ, USA).

### 2.10. Preparation of Washed Platelets 

Citrated whole blood was obtained from healthy donors according to a protocol approved by the Institutional Review Board of Chang Gung Memorial Hospital (Linkou, Taiwan) with the approval ID 202100952A3. The washed platelets were prepared as previously described with some modifications [42]. Briefly, citrated human blood was centrifuged at 670× *g* for 9 min to obtain platelet-rich plasma (PRP). After the addition of PGI2 (1 µM) to PRP, followed by centrifugation at 980× *g* for 10 min, platelet pellets were obtained. After washing twice with Tyrode’s buffer (137 mM NaCl, 2.65 mM KCl, 12 mM NaHCO_3_, 0.43 mM NaH_2_PO_4_, 2 mM CaCl_2_, 1 mM MgCl_2_, 5 mM glucose, 5 mM HEPES, pH 7.35) containing PGI_2_, washed platelets were re-suspended in Tyrode’s buffer to a final concentration of 3 × 10^8^ platelets/mL. The washed platelets were then subjected to functional assays as described. 

### 2.11. Platelet Aggregation Assay

A 96-well platelet aggregation assay was performed as described previously with some modifications [43]. Briefly, 100 μL of washed platelets (3 × 10^8^/mL) was placed into each well of a 96-well plate containing recombinant PDPN protein (0.8 μM), anti-PDPN antibody NZ-1 (320 nM), A1 aptamer (160 or 320 nM) or SYLC3 aptamer (160 or 320 nM). The 96-well plate was then placed into a plate reader for the measurement of light absorbance at 595 nm under 37 °C. The absorbance was recorded every 15 sec for a total of 16 min. The plate was vigorously shaken during the measurement interval. The percentage of aggregation at the endpoint was calculated with the degree of absorbance for Tyrode’s buffer, and the washed platelet was arbitrarily set to either 100% or 0% aggregation, respectively. The data represent the aggregation responses at 16 min after the initiation of the reaction.

### 2.12. Measurement of Circular Dichroism (CD)

The secondary structures of aptamers (10 μM, 5 mM MgCl_2_ in PBS) were analyzed using CD spectroscopy. The aptamers were denatured at 95 °C for 5 min and cooled on ice for at least 5 min before analysis. A J-815 CD Spectrometer (JASCO Corporation, Hachioji, Japan) was used to measure the CD spectra of the aptamers by placing the aptamer in a 0.1-cm path-length quartz cuvette and analyzing it under a constant flow of dry nitrogen. Scans were performed three times from 200 to 350 nm at 100 nm/min with a 1-s response time, 1-nm pitch, and 1-nm bandwidth. The CD spectrum of buffer (5 mM MgCl_2_ in PBS) was measured in the same manner and subtracted from the collected data.

### 2.13. Statistical Analysis

Data were expressed as the mean ± standard error of means (SEM). The unpaired student *t*-test was used for statistical analysis when appropriate. *p* < 0.05 was considered as statistical significance.

## 3. Results

### 3.1. Screening for Aptamers Recognizing PDPN

**Figure 1:**
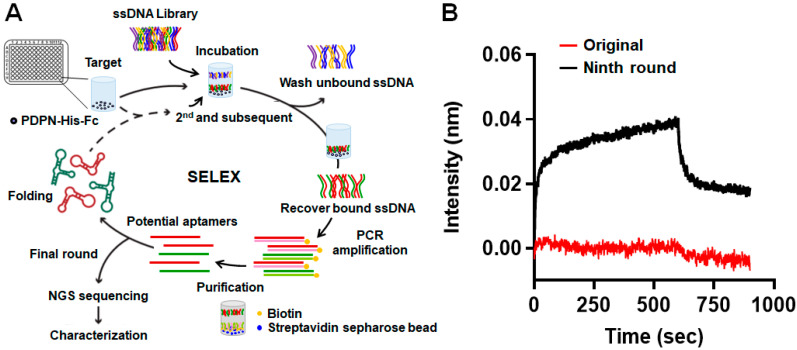
SELEX for the screening of the PDPN aptamer. (**A**) Schematic representation of the selection process of SELEX employed to identify PDPN aptamer. (**B**) The binding of PDPN (100 nM) to the initial aptamer library (original) or the ssDNA pool obtained from the ninth round of SELEX screening (Ninth round) was performed using the BLI assay. Representative binding intensity during the association and dissociation of PDPN to the initial aptamer library and the ssDNA pool coated on the chip are shown.

SELEX was performed to identify aptamers that recognized PDPN (Figure 1A). A synthetic ssDNA library was screened against PDPN through consecutive rounds of incubation, separation, and PCR amplification. After nine rounds of SELEX screening, the ssDNA pool was subjected to BLI assay to investigate whether there was an enrichment of ssDNA against PDPN (Figure 1B). As shown by the association and dissociation curves for the binding of PDPN to the initial aptamer library or the ssDNA pool after nine rounds of screening on the chip, we inferred that PDPN aptamers were enriched and presented in the ssDNA pool.

**Figure 2:**
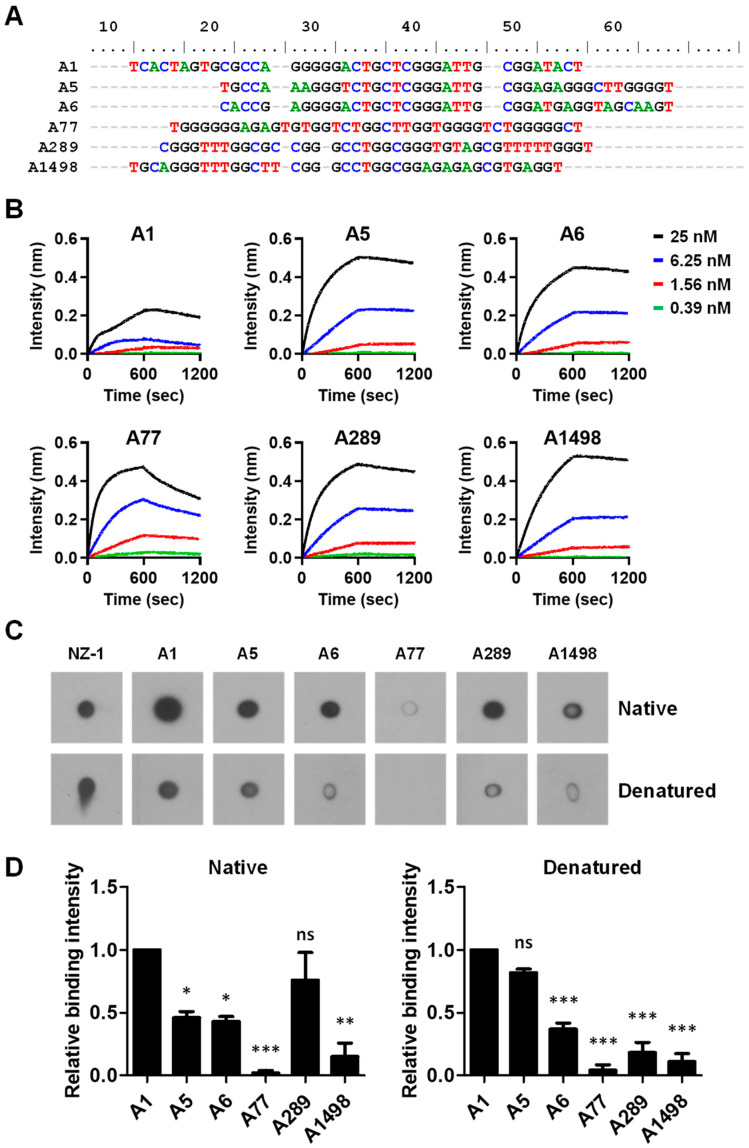
The characterization of putative PDPN aptamers. (**A**) The alignment of six representative aptamer sequences by using Bioedit software. (**B**) Aptamers were coated on the BLI chip and incubated with the indicated concentrations of PDPN. The dissociation constant K_d_ was calculated, as shown in Table 1. (**C**) The dot blot analysis for binding between PDPN and the six putative PDPN aptamers. PDPN (50 nM) was spotted on the nitrocellulose membrane followed by subsequent incubation with the indicated biotin-labeled aptamers (80 nM) and the streptavidin-conjugated HRP. The binding of the anti-PDPN antibody (NZ-1) to the PDPN spotted on the nitrocellulose membrane was used as a positive control. (**D**) Statistical analysis for the binding between PDPN and the six putative PDPN aptamers in dot blot analysis, as shown in panel (**C**). The binding intensity of A1 to PDPN was arbitrarily set as 1. The data represent the mean ± SEM of 2 to 3 independent experiments. *, *p* < 0.05; **, *p* < 0.001; ***, *p* < 0.0001; ns, no significance.

With evidence for the presence of PDPN aptamers in the ssDNA pool, NGS was performed to determine the compositions of the ssDNA pools. A total of 23,551 unique aptamer sequences were defined. Among the aptamer pools, 407 unique sequences had a frequency of ≥0.01%. An analysis of the aptamer sequences by alignment using the Bioedit software version 7.0.5.3 revealed that most of the aptamers shared consensus core sequences with highly diverse flanking sequences (Figure 2A). At least 6 different types of sequences were present in the ssDNA pool, represented by the aptamers A1, A5, A6, A77, A289, and A1498, respectively. A1, A5, and A6 were the top 3 aptamer sequences with frequencies of 68.35%, 9.40%, and 4.76%, respectively. The frequencies for A77, A289, and A1498 were not high and were only 0.09%, 0.03%, and 0.01% for the ssDNA pools, respectively (Table 1). BLI assay revealed that these aptamers bound to PDPN with a similar dissociation constant (Kd) in the nM scale (Figure 2B and Table 1). Dot blot assay revealed that these aptamers had better binding to native PDPN compared to the denatured form of PDPN (Figure 2C). A1 and A77 elicited the strongest and weakest binding to PDPN compared to the other aptamers, respectively (Figure 2D).

### 3.2. Analysis of the Putative PDPN Aptamers Structures

**Figure 3:**
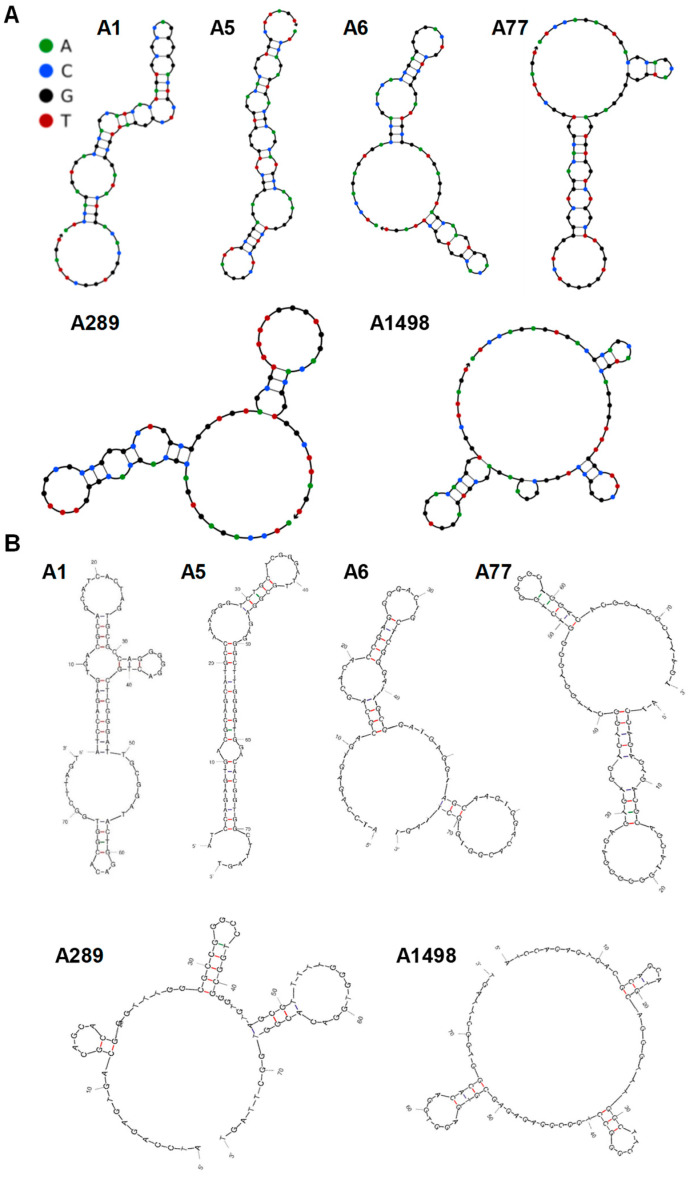
Schematic illustrations of the steric structures of the putative PDPN aptamers. (**A**,**B**). The sequences of the indicated PDPN aptamers were subjected to structural analyses using the structure prediction softwares NUPACK 4.0 (panel (**A**)) and mFold (panel (**B**)). Stem and loop structures were formed.

**Figure 4:**
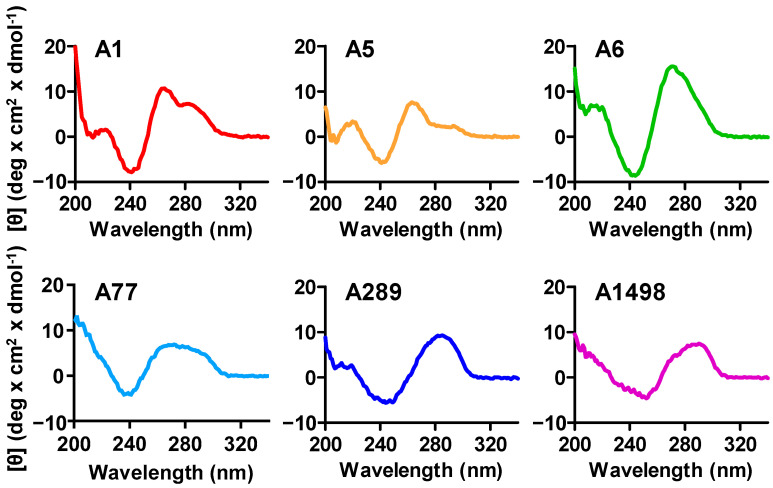
CD spectra of the six putative PDPN aptamers. The aptamers were denatured at 95 °C for 5 min and cooled on ice for at least 5 min before analysis. The CD spectra were measured using a J-815 CD Spectrometer (JASCO Corporation, Hachioji, Japan). The six aptamers displayed distinctive G-quadruplex patterns.

The secondary structures of the isolated aptamers, as predicted by NUPACK 4.0 or mFold, revealed that A1, A5, A6, and A77 shared similar long stem-and-loop structures, while A289 and A1498 formed large loop structures with several small loops (Figure 3A,B). The six PDPN aptamers (A1, A5, A6, A77, A289, and A1498) reported in this study were all G-rich, implying the possibility of G-quadruplex structures. To experimentally verify the secondary structures of the six PDPN aptamers, CD spectroscopy, which could be used to analyze DNA structures [44], was performed (Figure 4). A1, A5, A6, and A77 showed CD spectra with negative and positive peaks at approximately 240 and 260 nm, respectively. which corresponded to the spectrum of parallel-type G-quadruplexes [45,46]. A1, A5, and A6 all had small but distinct peaks at approximately 290 nm, which were in contrast to A77, which had broad peaks from 260 to 290 nm. This implied that the structure of A77 was distinctive from A1, A5, A6, and A77, despite them all forming parallel-type G-quadruplexes. On the other hand, A289 and A1498 showed negative and positive peaks at approximately 250–260 nm and 280–290 nm, respectively, which appeared to correspond with the spectrum of the DNA structure with antiparallel-type G quadruplexes or B-DNA helix [46,47,48,49]. The data were in accord with the NUPACK 4.0 and mFold predictions, which showed distinctive patterns for the structures of A289 and A1498.

### 3.3. Characterization of the PDPN Aptamer A1

**Figure 5:**
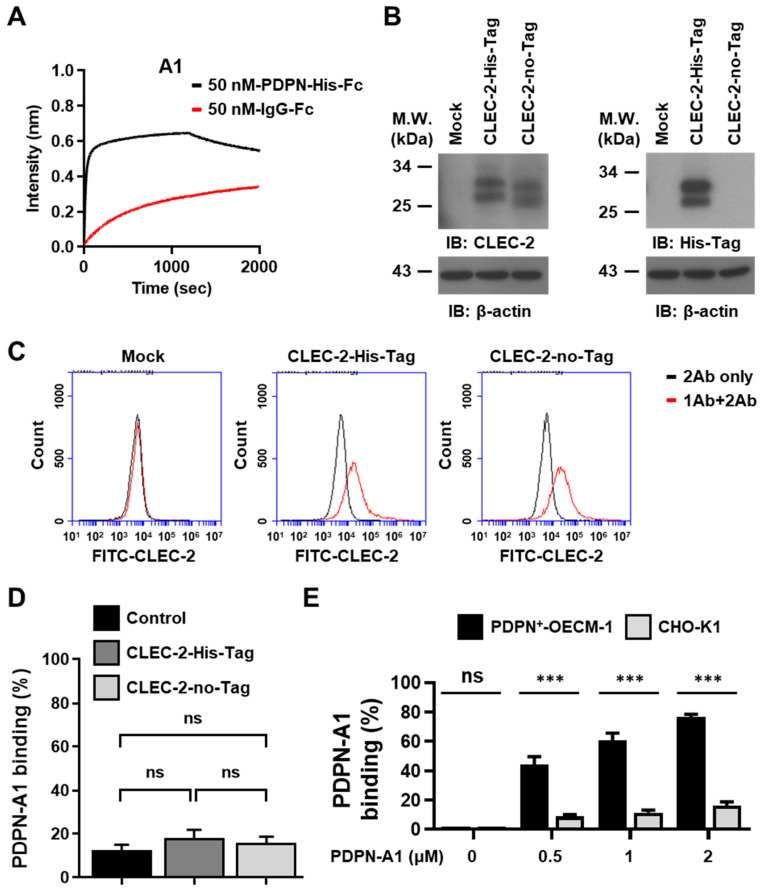
The binding specificity of A1 aptamer to PDPN. (**A**) PDPN-His-Fc or IgG-Fc (50 nM) was coated on the BLI chip and incubated with the A1 aptamer for BLI assay. The association and dissociation curves for the binding of the A1 aptamer with the recombinant PDPN and IgG-Fc are shown. (**B**) Western blot analysis was performed for the lysates from HEK293T cells transfected with the indicated expression plasmid. Protein expression was detected by using the anti-CLEC-2 antibody (left panel) or the anti-His-Tag antibody (right panel). (**C**) Flow cytometry analysis of CLEC-2 expression on the cell surface. The HEK293T cells were transfected with the indicated plasmids encoding CLEC-2 protein with or without the His-Tag sequence. At 48 h after transfection, the cells were subjected to flow cytometry analysis using the primary mouse anti-human CLEC-2 antibody and the secondary FITC-conjugated goat anti-mouse antibody. Representative histograms are shown. (**D**) HEK293T cells were transfected with the plasmids encoding the indicated proteins. At 48 h after transfection, the cells were incubated with the FAM-conjugated A1 aptamer (1 μM). The percentages of cells with A1 binding were analyzed by flow cytometry. The data represent the mean ± SEM of three independent experiments. (**E**) The indicated cell lines were incubated with different concentrations of the FAM-conjugated A1 aptamer. Flow cytometry was performed to determine the levels of A1 binding on the cell surface. The data represent the mean ± SEM of three independent experiments. ns, no significance; ***, *p* < 0.001.

Based on the stronger binding of the A1 aptamer to the native and denatured PDPN than the other aptamers, the A1 aptamer was selected for further characterization. As revealed by the BLI assay, there was no interaction between A1 and Fc-IgG, ruling out the A1 that was bound to the Fc region of the recombinant PDPN (Figure 5A). To investigate whether A1 was bound to the His-Tag in the recombinant PDPN protein, expression plasmids encoding CLEC-2 with or without His-Tag were constructed. His-Tag was placed in the C-terminus of the protein where the extracellular domain was located. The CLEC-2 protein was detectable in the lysates of HEK293T cells transiently transfected with the expression plasmids of pcDNA4-hCLEC-2-His-Tag or pcDNA4-hCLEC-2-no-Tag (Figure 5B, left panel). His-Tag protein was only detectable in the lysates of HEK293T cells expressing pcDNA4-hCLEC-2-His-Tag, as revealed by Western blotting using the anti-His-Tag antibody (Figure 5B, right panel). Both recombinant CLEC-2 proteins with or without His-Tag were expressed on the cellular surface, as revealed by the flow cytometry analysis (Figure 5C). A1 did not elicit any binding to CLEC-2 with or without His-Tag when compared to the control cells (Figure 5D), implying that His-Tag was not the binding target of A1.

To further delineate the specific binding of A1 to PDPN, OECM-1 cells expressing PDPN (PDPN^+^-OECM1) without any Tag-sequences and the CHO-K1 cells without PDPN expression were incubated with the FAM-conjugated A1 aptamer. This was followed by flow cytometry analysis (Figure 5E). A1 aptamer bound to the PDPN^+^-OECM1 cells in a concentration-dependent manner. On the other hand, A1 did not elicit binding activity toward the CHO-K1 cells (*p* < 0.05). These data indicated that A1 aptamer recognized PDPN expressed on the cell surface. 

### 3.4. Quantitative Detection of PDPN by Aptamer A1

**Figure 6:**
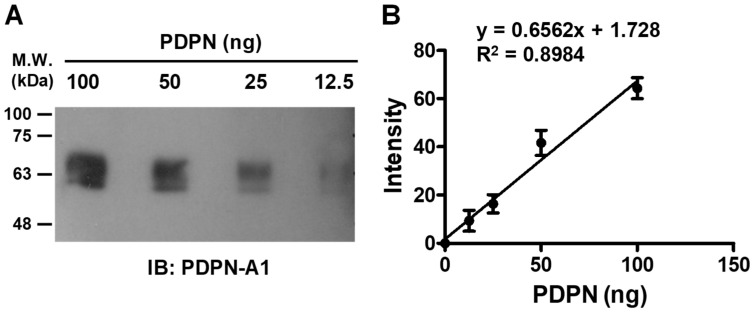
The quantitative detection of PDPN by the aptamer A1. (**A**) The indicated amounts of PDPN protein were subjected to Western blot analysis. The biotin-labeled aptamer A1 and the streptavidin-conjugated HRP were used to detect PDPN protein by ECL. (**B**) The linear correlations of the signal intensities and the amounts of PDPN present in the samples were performed. The data represent the mean ± SEM of four independent experiments. The linear correlation equation and the R^2^ value are shown.

To delineate whether aptamer A1 can be used as a “chemical antibody” for the detection of PDPN in Western blot analysis, various amounts of recombinant PDPN protein were fractionated on an SDS-PAGE gel, and the proteins were transferred to the PVDF membrane. After incubating the membrane with biotin-labeled aptamer A1 followed by streptavidin-conjugated HRP, PDPN proteins were detectable by ECL (Figure 6A). A linear correlation (R^2^ = 0.8984) between the intensity of the signal and the amounts of PDPN protein present in the samples was observed (Figure 6B). These data indicated that the PDPN aptamer A1 was applicable for quantitatively detecting PDPN. 

### 3.5. Aptamer A1 Interfering with the Interaction between PDPN and CLEC-2

**Figure 7:**
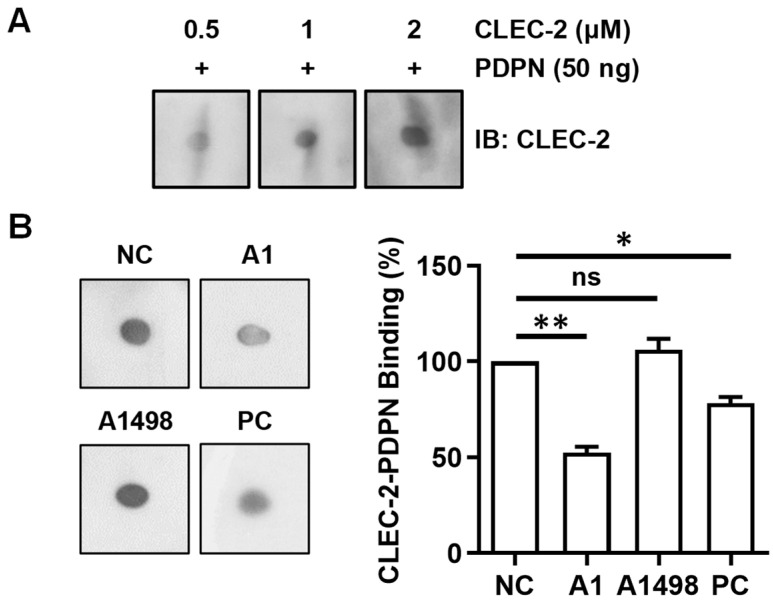
A1 aptamer interferes with PDPN/CLEC-2 interactions. (**A**) The recombinant PDPN protein was spotted on the nitrocellulose membrane and incubated with the indicated concentrations of the CLEC-2 protein. The binding of CLEC-2 to the spotted PDPN was detected using the anti-CLEC-2 antibody followed by the secondary antibody. ECL was performed to detect the signal. (**B**) PDPN was spotted on the nitrocellulose membrane. The A1 aptamer (A1), A1498 aptamer (A1498), or anti-PDPN antibody NZ-1 (PC) and the CLEC-2 proteins were incubated with the membrane. The amount of CLEC-2 binding to PDPN was determined by incubating the membrane with the anti-CLEC-2 antibody followed by the secondary antibody. ECL was performed to detect the signal. Quantification was performed using ImageJ. The amount of CLEC-2 binding to PDPN without aptamer or antibody (NC group) was arbitrarily set to 100%. The data represent the mean ± SEM of three independent experiments. *, *p* < 0.05; **, *p* < 0.01; ns, no significance.

A dot blot assay was first established to demonstrate binding between PDPN and CLEC-2. Recombinant PDPN proteins were spotted on the nitrocellulose membrane and incubated with different concentrations of CLEC-2 proteins in aqueous solution (Figure 7A). The binding of CLEC-2 on PDPN was then revealed using the anti-CLEC-2 antibody. CLEC-2 elicited a concentration-dependent binding to PDPN, implying that the dot blot assay could be used to analyze the effects of A1 aptamer on the interaction of PDPN and CLEC-2. 

To delineate whether A1 aptamer could interfere with PDPN/CLEC-2 binding, A1 and CLEC-2 were co-incubated with PDPN spotted on the nitrocellulose membrane in the dot blot assay (Figure 7B). The A1 aptamer resulted in a 52.7 ± 2.8% decrease in PDPN/CLEC-2 binding, which appeared to be better than the anti-PDPN antibody NZ-1 (PC group, 78.1 ± 3.5%) at a concentration of 3.0 μM. In contrast, the A1498 aptamer did not interfere with the interaction between PDPN and CLEC-2. These data indicated that the A1 aptamer could effectively interfere with the interaction between PDPN and CLEC-2. 

### 3.6. Aptamer A1 Inhibiting PDPN-Induced Platelet Aggregation

**Figure 8:**
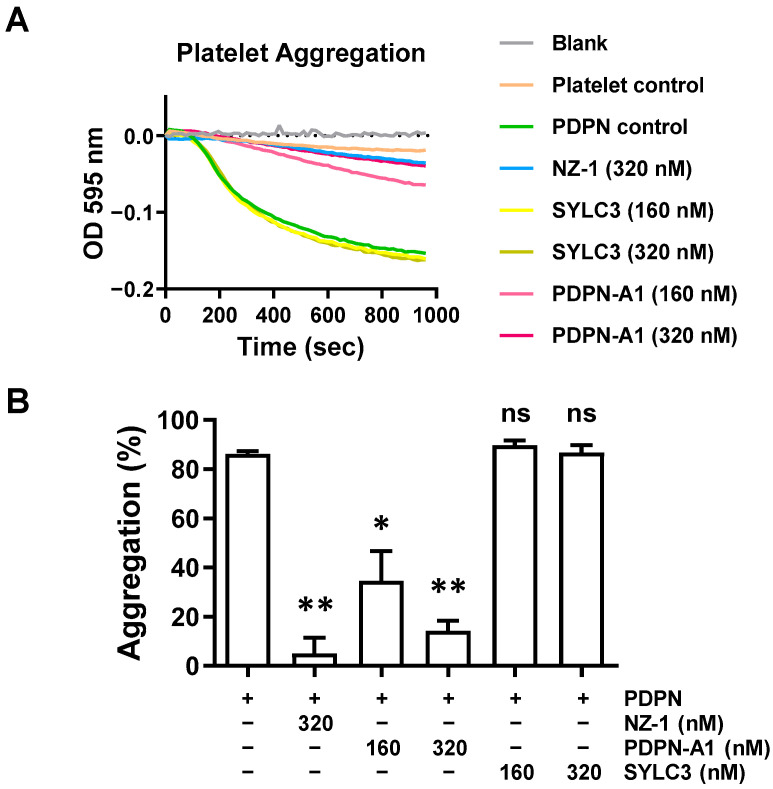
Aptamer A1 inhibits PDPN-induced platelet aggregation. (**A**,**B**) PDPN-induced platelet aggregation was performed using the 96-well plate method. The kinetics of platelet aggregation in the presence or absence of the indicated aptamers or anti-PDPN antibody NZ-1 were determined by the continuous measurement of light absorbance at OD595 nm using the SpectraMax Plus 384 Microplate Reader (Marshall Scientific, Hampton, NH, USA). Representative platelet aggregation traces for the indicated treatments are shown (panel (**A**)). The degree of aggregation at the end of the assay was determined. The data represent the mean ± SEM of three independent experiments (panel (**B**)). *, *p* < 0.05; **, *p* < 0.01; ns, no significance.

To explore whether A1 inhibited PDPN-induced platelet aggregation, a 96-well platelet aggregation assay was first established according to a previous study [43]. The degree of platelet aggregation was monitored by measuring the light absorbance at 595 nm (Figure 8A). PDPN caused platelet aggregation and the change in light absorbance at OD595 nm. PDPN-induced platelet aggregation was inhibited by the anti-PDPN antibody NZ-1 and the A1 aptamer. In contrast, the SYLC3 aptamer [50] that targeted the epithelial cell adhesion molecular (EpCAM) did not elicit any effect on PDPN-induced platelet aggregation (Figure 8B). The A1 aptamer, thereby, was able to bind PDPN and interfere with its binding, with CLEC-2 leading to the inhibition of PDPN-induced platelet aggregation.

## 4. Discussion

In this study, an aptamer A1 that recognized PDPN was obtained by screening ssDNA pools using the SELEX method. The A1 aptamer bound PDPN and interfered with PDPN/CLEC-2 interactions and PDPN-induced platelet aggregation. This study represented the first report to define a functional PDPN aptamer with the potential to interfere with platelet–cancer cell interactions mediated by the binding of PDPN to the platelet CLCE-2. 

Antibodies are widely used in biomedical applications and serve as biosensors for protein detection or blocking protein interactions [51]. However, the generation and production of antibodies are usually time-consuming and difficult to handle and store. With the intrinsic nature of nucleotides, aptamers provide several advantages over antibodies [52,53]. Aptamers are more stable than antibodies. Aptamers display low to no immunoreactivity, which is important for therapeutic applications. Aptamers can be produced by chemical synthesis with low batch-to-batch variability and high purity. The small sizes of aptamers make it easier for them to modify and reach targets inside the cells than antibodies, allowing for applications in cellular and imaging analyses. These advantages present the great impacts of aptamers on research, diagnostics, and therapy [54,55]. Aptamers have been proposed as a substitute for antibodies in various applications. For example, the aptamers REG1 [56] and NU172 [57] could inhibit the activities of FIXa and thrombin, respectively. The aptamer NU172 has been tested as an anticoagulant in clinical trials [58]. Aptamers targeting the PD-1/PD-L1 axis have been shown to elicit anti-tumor effects [59,60]. Aptamer-based biosensors for protein and peptide detection have been widely developed [61,62,63]. The A1 aptamer, as reported in this study, represents a new tool for analyzing PDPN expression by flow cytometry, quantitatively detecting PDPN proteins by Western blot analysis, and the functional interference of PDPN-initiated CLEC-2 signaling. It also presents an opportunity to develop a method to detect PDPN expression in clinical specimens, such as liquid biopsy and tissues. 

Whether the aptamer elicits a specific interaction with the target protein is crucial for its application [64], particularly when the recombinant proteins with Tag sequences are used during the screening process. Several lines of evidence support A1 binding specifically to PDPN but not the Fc- or His-Tag sequences in the recombinant PDPN protein. The small dissociation constant (K_d_) of 1.3 ± 1.2 nM between A1 aptamer and PDPN implies a strong and specific interaction between A1 and PDPN. The results of the BLI assay rule out the Fc region of recombinant PDPN protein as the binding site for the A1 aptamer. Flow cytometry analyses using CLEC-2 proteins with or without His-Tag further indicate that A1 binds to PDPN but not to the His-Tag region of the recombinant PDPN protein. Notably, A1 aptamer can bind to PDPN without any Tag sequences expressing on the cell surface. These findings support the specific interaction of A1 with PDPN and imply that the A1 aptamer binds to PDPN through recognizing of PDPN. However, the presence of other sequences associated with recombinant protein generation is not supported. The specific region of A1 that is important for its binding to PDPN needs to be elucidated.

Analyses of the secondary structures by NUPACK 4.0 and mFold indicate that the isolated aptamers all form stem-and-loop structures. In contrast to the long stem-and-loop structures of other aptamers, A289 and A1498 display distinctive large loop structures with several small loops. CD spectra also show distinctive patterns of A289 and A1498 with a negative peak at ~250 nm and a positive peak at ~280 nm, in contrast to the other aptamers showing a negative peak at ~240 nm and a positive peak at ~260 nm. This implies that the two groups of aptamers may have different secondary structures [44,46]. It is likely that the two groups of aptamers have different binding properties and bind at different sites of PDPN. 

BLI analysis reveals that isolated aptamers have similar binding affinity (K_d_) values, but the bindings of these aptamers to native and denatured PDPN in the dot-blot assay are distinctive. A1 elicits the strongest binding, and A77 elicits the weakest binding to either native or denatured PDPN. Similar observations have been reported in previous works [65,66,67]. For example, the anti-HER3 aptamers target MCF7 cells effectively by using radiolabeling techniques [68]. However, no binding was observed when the same aptamers were used in flow cytometry [69]. It is likely that specific aptamers act differently in various techniques used and under various assay conditions. The pH, aptamer folding, and buffer system may all contribute to the complexity of the binding between the aptamer and the targeted proteins using different techniques. In this study, BLI assay was performed with the aptamers immobilized on the chip, while dot-blot assay was performed with the PDPN protein spotted on the nitrocellulose membrane. The steric differences for the aptamer and PDPN proteins in these two assay conditions may contribute to the different binding characteristics of the specific aptamers to PDPN, which may partly explain the different binding properties of A1 and A77 in BLI and dot-blot assays. The studies by us and others highlight the importance of choosing an appropriate method to evaluate the binding properties of putative aptamer candidates. A comprehensive screening among different methods is preferred to test the binding ability and consistency of the potential aptamers to the target protein.

PDPN is currently the only known endogenous ligand of CLEC-2, a platelet receptor barely found in other blood cells [12]. Activation of CLEC-2 signaling does not affect physiological hemostasis, as demonstrated by the CLEC-2-deficient platelets, which normally respond to the platelet agonists of collagen, ADP, U46619, and PAR-4 peptide [19,70]. In addition to its involvement in cancer progression [71], recent studies indicate that the PDPN/CLEC-2 signaling axis is crucial in various physiological and pathological conditions such as blood cell generation, inflammatory response, and thrombus formation [25,72]. For example, PDPN/CLEC-2 interaction regulates blood-lymphatic vessel separation [73], wound healing [74], efficient T-cell priming [75,76], the maintenance and integrity of high endothelial venules in lymph nodes and cerebrovascular patterning [77,78], megakaryocyte proliferation, proplatelet formation [79]. With the capability to bind PDPN and interfere with PDPN/CLEC-2 interactions, the A1 aptamer has the potential to modulate PDPN/CLEC-2 signaling linking to various pathological conditions. While the inhibitory effects of A1 aptamer on PDPN/CLEC-2 interactions are comparable to the anti-PDPN antibody NZ-1, it is worth exploring further the functions of A1 aptamer in various cellular and molecular systems and animal disease models.

There are still limitations of this study. The A1 aptamer-based biosensor or assay may not have enough sensitivity to detect a minute amount of target protein. This may restrict its use in PDPN detection. Higher concentrations of A1 aptamer may be required to overcome this issue. In physiological conditions, ssDNA may interact with various proteins [80,81]. The binding regions of the protein are positively charged via electrostatic complementarity to compensate for the negatively charged phosphate backbone in the oligonucleotide. In addition, unpaired nucleotides also play important roles in protein recognition [82]. Thus, identification of structural characteristics of A1 aptamer is required to further understand the interactions between A1 and PDPN. This may help design a better PDPN aptamer to improve its specificity and detection limit in biomedical applications.

Taken together, we defined for the first time an aptamer that binds PDPN and interferes with the PDPN/CLEC-2 signaling axis. The A1 aptamer has the potential to be developed as a biomedical tool for the analysis of PDPN expression and as an effective pharmacological agent in the inhibition of cancer metastasis and various pathological conditions connected with the activation of CELC-2 by PDPN.

## Figures and Tables

**Table 1:** The characteristics of putative PDPN aptamers.

Aptamer	Sequences	Frequency (%)	K_d_ (nM) ^a^
A1	5′-ATCCAGAGTGACGCAGCATCACTAGTGCGCCAGGGGGA CTGCTCGGGATTGCGGATACTGGACACGGTGGCTTAGT-3′	68.35	1.3 ± 1.2
A5	5′-ATCCAGAGTGACGCAGCATGCCAAAGGGTCTGCTCGGG ATTGCGGAGAGGGCTTGGGGTGGACACGGTGGCTTAGT-3′	9.40	0.2 ± 0.1
A6	5′-ATCCAGAGTGACGCAGCACACCGAGGGGACTGCTCGGG ATTGCGGATGAGGTAGCAAGTGGACACGGTGGCTTAGT-3′	4.76	0.2 ± 0.0
A77	5′-ATCCAGAGTGACGCAGCATGGGGGGAGAGTGTGGTCTG GCTTGGTGGGGTCTGGGGGCTGGACACGGTGGCTTAGT-3′	0.09	1.7 ± 0.1
A289	5′-ATCCAGAGTGACGCAGCACGGGTTTGGCGCCGGGCCTG GCGGGTGTAGCGTTTTTGGGTGGACACGGTGGCTTAGT-3′	0.03	0.5 ± 0.0
A1498	5′-ATCCAGAGTGACGCAGCATGCAGGGTTTGGCTTCGGGC CTGGCGGAGAGAGCGTGAGGTGGACACGGTGGCTTAGT-3′	0.01	0.4 ± 0.0

^a^ The data represent the mean ± SEM of three independent experiments.

## Data Availability

All relevant data are included in the paper.
